# Multiple Dermal Abscesses by *Trichophyton rubrum* in an Immunocompromised Patient

**DOI:** 10.3389/fmed.2019.00097

**Published:** 2019-05-06

**Authors:** Frédéric Toussaint, Michael Sticherling

**Affiliations:** Department of Dermatology, University Hospital Erlangen, Friedrich-Alexander-University Erlangen-Nürnberg (FAU), Erlangen, Germany

**Keywords:** dermatophytosis, abscess, *Trichophyton rubrum*, immunosuppression, fungal infection

## Abstract

We report the case of a 52-year-old man who presented with a 10 year history of multiple nodules with purulent drainage on the upper extremities. Several attempts of treatment with oral antibiotics had been unsuccessful. A skin biopsy specimen showed a dermal abscess with branched septate hyphae. A mycological culture of pus and of the biopsy specimen revealed *Trichophyton rubrum*. Deeper dermatophytosis presenting as dermal abscesses is a rare disease which occurs normally in immunocompromised conditions. Our patient was on immunosuppressive therapy with methylprednisolone and azathioprine because of inflammatory demyelinating polyneuropathy and presented with extensive abscesses. In cases of dermal abscesses it is important to not only consider bacterial but also fungal infections as underlying cause.

## Introduction

Dermatophyte infection is a common disease which is usually limited to the stratum corneum, nails, and hair. However, in some cases dermatophytes cause invasive infections beyond the keratinized layer of the skin. Invasive dermatophytic infection can be classified in three forms: Majocchi' s granuloma, also called nodular granulomatous perifolliculitis, deeper dermal dermatophytosis, and the very rare form of invasive disseminated dermatophytosis with dissemination to internal organs (1). We present a case of deeper dermal dermatophytosis in form of multiple dermal abscesses caused by *Trichophyton rubrum* in an immunosuppressed patient.

## Case Presentation

A 52-year-old obese (100 kg, 1.80 m) Caucasian man presented in January 2017 with multiple nodules with purulent drainage on the upper extremities persisting for more than 10 years. Several attempts of treatment with oral antibiotics had been unsuccessful. The number of nodules was increasing over the time. He had a medical history of chronic inflammatory demyelinating polyneuropathy diagnosed in 2000. Therefore, he was on immunosuppressive therapy with methylprednisolone 20 mg per day and azathioprine 200 mg per day.

**Figure 1:**
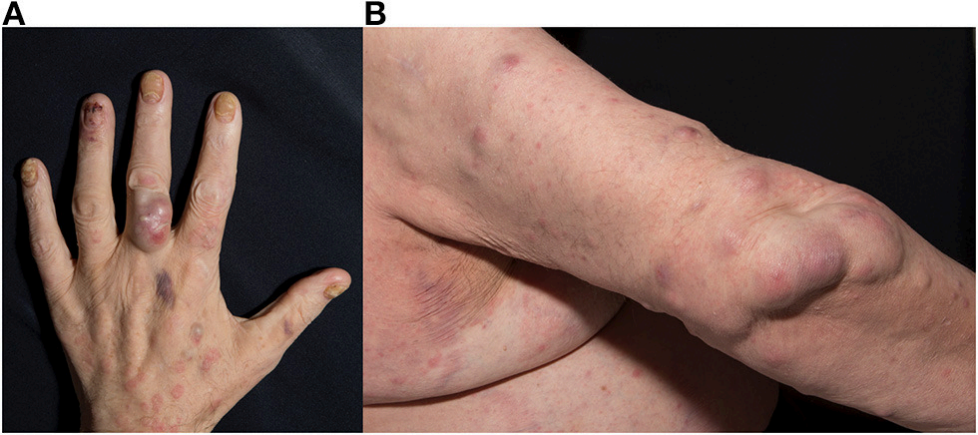
**(A,B)** Fluctuant nodules on the proximal phalanx of the left middle finger and the right arm.

**Figure 2:**
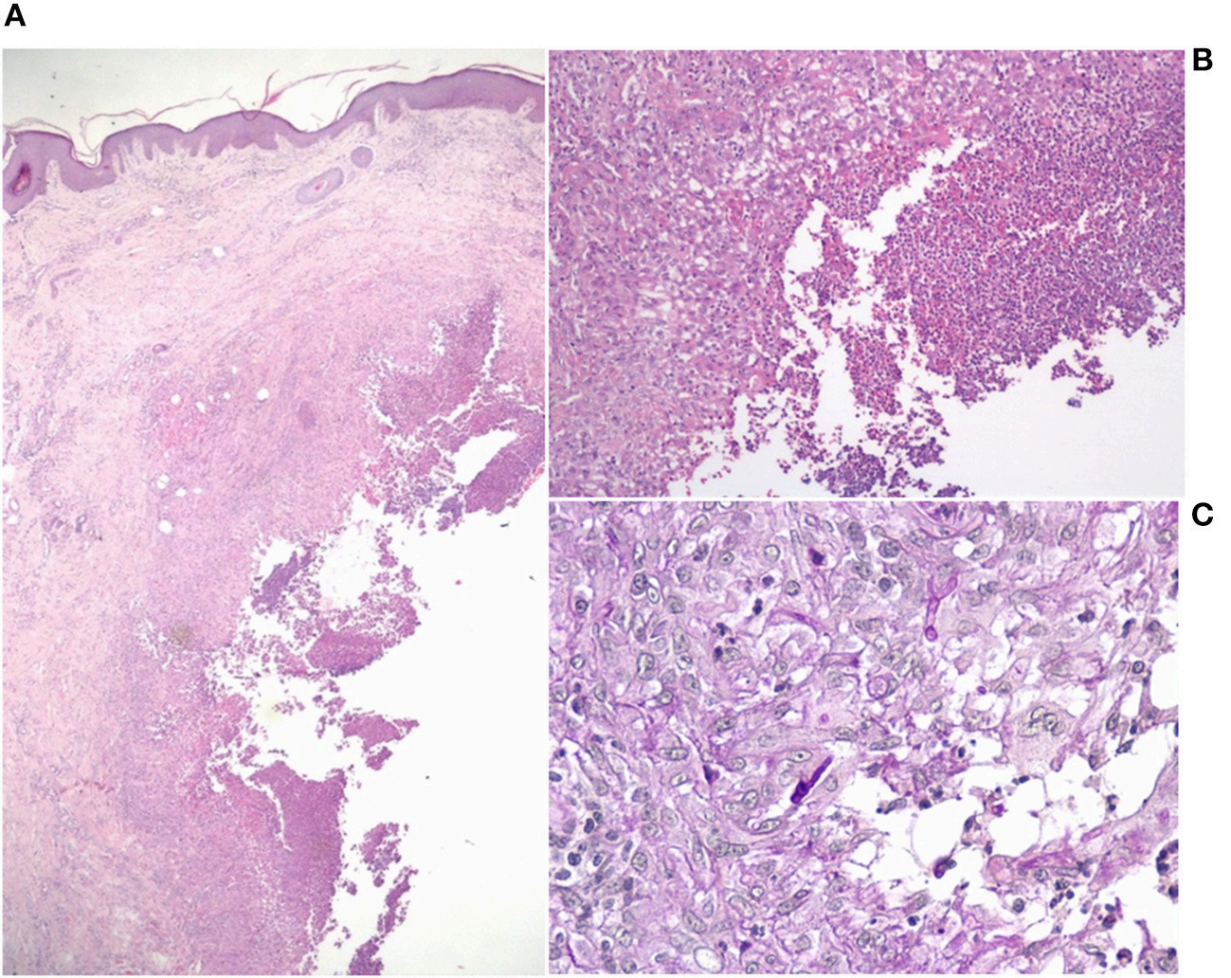
Histopathological finding of a nodule from the right forearm. **(A)** Dermal abscess with massive neutrophils in the center [hematoxylin and eosin (HE) staining, original magnification x20]. **(B)** Macrophages in the border area [HE staining, original magnification x100]. **(C)** Branched septate hyphae in the transition zone between granulomatous inflammation and abscess [Periodic Acid Schiff (PAS) staining, original magnification x400].

Additionally, he was on medication with acetylsalicylic acid after a myocardial infarction in 2010 and antihypertensives. Physical examination showed a violaceous firm nodule with purulent drainage over the proximal phalanx of the left middle finger (Figure 1A) and up to 20 reddish to violaceous firm nodules up to 4 × 2 cm in size on the right arm (Figure 1B). Additionally, there were extensive well-demarcated, erythematous macules with scaly borders on the chest. All finger- and toenails showed onychodystrophy and yellowish discoloration. Furthermore, physical examination revealed an enlarged lymph node in the left axilla. Abdominal ultrasound revealed a slight hepato- and splenomegaly. Biochemical examination showed an elevated white blood cell count (16.300 /μl) with relative lymphocytopenia, low hemoglobin (9.9 g/dl) with iron deficiency and elevated HbA1c (7.9%). Other routine laboratory tests were unremarkable. Screening for human immunodeficiency virus and tuberculosis was negative. Direct microscope examination by potassium hydroxide (KOH) preparation of scales from a lesion of the chest, of nail scrapings and of pyogenic fluid of a nodule showed each branched septate hyphae. Fungal culture of each of the above mentioned specimens revealed *T. rubrum*. Bacterial cultures were negative. A biopsy specimen of a nodule from the right forearm showed a dermal abscess with massive neutrophils in the center and macrophages in the border area (Figures 2A,B). The Periodic Acid Schiff (PAS) staining showed branched septate hyphae in the transition zone between granulomatous inflammation and abscess (Figure 2C). Fungal culture of the biopsy specimen also showed *T. rubrum*. Culture for non-tuberculous mycobacteria was negative. Furthermore, a biopsy specimen from the erythematous macula on the chest showed the histopathologic picture of tinea superficialis with branched septate hyphae in PAS staining. The patient was diagnosed as deeper dermatophytosis by *T. rubrum* presenting in form of multiple dermal abscesses. Treatment was started with oral itraconazole 200 mg per day and local application of ciclopirox twice a day. The patient did not show up for follow up and medication was only unsteadily taken. Four months later the patient presented again in our department with now multiple well-demarcated erythematous scaly plaques on the trunk and growing size of the abscesses on the right elbow. He had presented to an office-based doctor, where a drug eruption was assumed, why treatment with itraconazole was stopped. However clinical examination and biopsy specimen of the plaques revealed tinea superficialis. Nevertheless, a treatment change to griseofulvin 500 mg twice a day was conducted. Again, the patient took the medication only unsteadily which resulted in an improvement of the clinical outcome but no cure with nodules still present after 6 months.

## Discussion

Deeper dermatophytosis is rare and most of the patients diagnosed with invasive dermatophytosis have underlying innate or acquired immunodeficiency such as CARD9 deficiency, immunosuppressive therapy because of solid organ transplant or autoimmune disease, hematological malignancies like myelodysplastic syndrome, leukemia and lymphoma, or HIV infection (2). In our presented case the patient was on azathioprine and methylprednisolone because of a chronic inflammatory demyelinating polyneuropathy. As additional immunosuppressive comorbidity the patient suffered from steroid-induced diabetes mellitus. In most cases of invasive dermatophytosis *T. rubrum* is verified, but also other species like *T. violaceum, T. mentagrophytes, M. canis, T. verrucosum*, and *T. ferrugineum* were found in cases of deeper dermatophytosis in form of dermal abscesses (3). While for deep dermatophytosis like Majocchi' s granuloma rupture of infected hair follicles or direct invasion from affected epidermis into the dermis are described (4), the portal of entry of dermatophytes in cases of deeper dermatophytosis like dermal abscess is less clear. In most of the cases of dermatophyte abscess a preexisting superficial dermatophytosis on the same site is reported (3). However, there are also cases with no preexisting superficial dermatophytosis described (5). In our case superficial dermatophytosis existed on the trunk, but not on the site of abscesses on the right arm and left middle finger. Additionally, the biopsy specimen of a nodule from the right forearm showed no epidermal involvement or fungal infection of hair follicules. The patient had onychomycosis in all finger- and toenails, so that one possible mechanism of dissemination and entry may be through scratching. Another hypothesis being discussed is the dissemination by lymphatic or heamatogenous spread (6). Dermatophytes are keratinophilic organisms which normally stay limited to keratinized structures. However, not only *in vitro* but also *in vivo* dermatophytes have been shown to be able to grow in non-keratinous tissue (7). The environment in the epidermis is more acid than in the dermis but cellular destruction and inflammation reactions with increased acid mucopolysaccharides may provide the pH requirements for dermatophytes (4). Also morphological changes like conversion to yeast-like forms, which may help to survive in other tissues than the epidermis, are reported (8). In an immunocompetent host, factors like the physical barrier, antimicrobial peptides and the innate and specific immune system prevent invasion of dermatophytes. Especially the cell mediated immunity is essential for fighting dermatophyte infection (9). In our patient the combined immunosuppressive therapy with methylprednisolone and azathioprine compromises severely the cell mediated immunity, providing a possible explanation for the unsatisfying course. In conclusion, in cases of dermal abscesses it is important to not only consider bacterial but also fungal infections as underlying cause. Especially in cases of long persisting abscesses with antibiotic treatment failure, mycological examinations should be taken. A hint for deeper dermatophytosis could be an existing superficial tinea or onychomycosis. In addition, particularly in immunocompromised patients, superficial tinea or onychomycosis has to be treated consequently in order to avoid deeper invasion.

## Patient Consent

Written informed consent was obtained from the participant for the publication of this case report and any potentially-identifying images/information.

## Author Contributions

FT contributed by drafting the work and MS by revising it critically. Both authors made substantial contributions to the conception or design of the work, made final approval of the version to be published and agree to be accountable for all aspects of the work in ensuring that questions related to the accuracy or integrity of any part of the work are appropriately investigated and resolved.

### Conflict of Interest Statement

The authors declare that the research was conducted in the absence of any commercial or financial relationships that could be construed as a potential conflict of interest.
